# Intraperitoneal administration of fosfomycin, metronidazole, and granulocyte-macrophage colony-stimulating factor in patients undergoing appendectomy is safe: a phase II clinical trial

**DOI:** 10.1038/s41598-019-43151-4

**Published:** 2019-04-30

**Authors:** Siv Fonnes, Barbara Juliane Holzknecht, Magnus Arpi, Jacob Rosenberg

**Affiliations:** 1 Centre for Perioperative Optimisation, Department of Surgery, Herlev and Gentofte Hospital, University of Copenhagen, Herlev Ringvej 75, DK-2730 Herlev, Denmark; 2 Department of Clinical Microbiology, Herlev and Gentofte Hospital, University of Copenhagen, Herlev Ringvej 75, DK-2730 Herlev, Denmark

**Keywords:** Coeliac disease, Phase II trials

## Abstract

We aimed to investigate the safety of intraperitoneal administration of the combination of fosfomycin, metronidazole, and recombinant human granulocyte-macrophage colony-stimulating factor (rhGM-CSF) in patients undergoing appendectomy. We conducted a prospective phase II clinical trial in 14 otherwise healthy men suffering from uncomplicated appendicitis. After appendectomy, the trial treatment was administered intraperitoneally and left in the abdominal cavity. Trial treatment consisted of 4 g fosfomycin, 1 g metronidazole, and 50 µg rhGM-CSF in a total volume of 500 ml. Safety was evaluated through white blood cell count where a toxic effect was predefined. We evaluated harms and adverse events, repeated biochemical markers, vital signs, and length of stay. White blood cell count did not drop below the toxic range. The recorded harms were dizziness, discomfort when breathing deeply, no flatus, and bloating. Adverse events included three patients with diarrhoea after discharge and one patient with a hypotensive episode. No serious adverse events or infectious complications occurred. Intraperitoneal administration of fosfomycin, metronidazole, and rhGM-CSF was safe in otherwise healthy men undergoing laparoscopic appendectomy. There were some possible harms and adverse events but we were unable to assess if they were related to anaesthesia, surgery, or the trial treatment.

## Introduction

Peritonitis is defined as inflammation involving the peritoneum. A common type is secondary peritonitis which occurs due to an intraabdominal surgically treatable source, e.g. perforation of the gastrointestinal tract, intestinal ischemia, or after surgery^1^. The severity of secondary peritonitis varies. Patients with an appendicular source of peritonitis have a low mortality of 0.1%^2^, whereas up to 50% of patients died in cohorts consisting of elderly and critically ill patients with severe intraabdominal infection^3^. The current treatment of secondary peritonitis consists of a combination of surgical intervention (source control), and relevant antimicrobial therapy according to the origin of the contamination^4^.

Our scope is to improve the treatment of peritonitis by attacking the peritonitis from different angles. Our treatment regimen consisted of the antimicrobial agents fosfomycin and metronidazol, and recombinant human granulocyte-macrophage colony-stimulating factor (rhGM-CSF) in combination with source control. Fosfomycin has bactericidal effects on Gram-positive and Gram-negative aerobic bacteria^5^ and metronidazole is widely used for anaerobic bacteria commonly seen in abdominal infections^4^. rhGM-CSF is a cytokine that promotes the growth, maturation, and activity of macrophages and neutrophil granulocytes^6–8^. It has previously been given subcutaneously to patients suffering from intraabdominal sepsis, where it shortened time to improvement and decreased the risk of infectious complications^9^. However, previous studies administration rhGM-CSF intraperitoneally or intravenously have reported a transitory drop in leukocytes^10–13^. Intraperitoneal administration achieves high concentrations at the site of the infection. Intraperitoneal treatment was recommended by a Cochrane review in patients suffering from peritonitis due to peritoneal dialysis, because it seemed superior to intravenously administered antibacterial agents^14^. All the drugs used in this trial had previously been given intraperitoneally individually^10,15–19^. We have shown that the combination of the drugs is stable *in vitro* and has maintained antibacterial activity^20^.

We aimed to evaluate the safety of intraperitoneal administration of the combination of fosfomycin, metronidazole, and rhGM-CSF in patients undergoing laparoscopic appendectomy for uncomplicated acute appendicitis in a phase II clinical trial. We evaluated safety primarily through of the white blood cell count (WBC).

## Results

**Figure 1:**
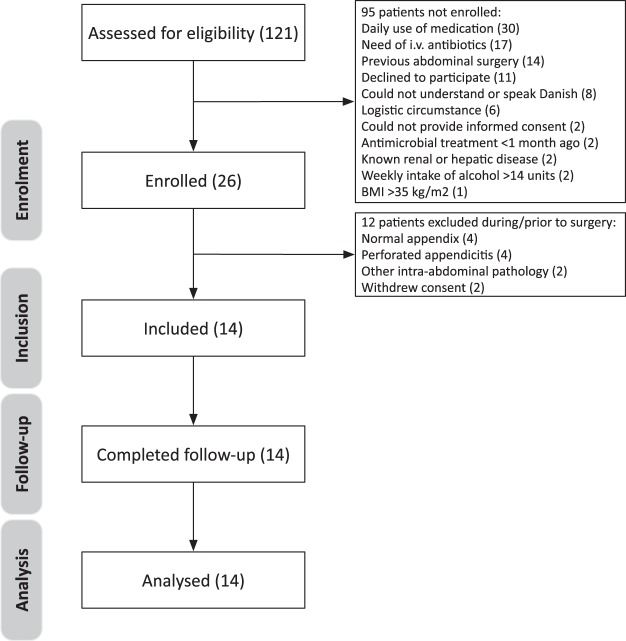
Flowchart of the screened, enrolled, and included patients in the trial.

The trial was initiated on February 24, 2017. The screening process is depicted in Fig. 1. We included 14 patients. The last day of follow-up was December 7, 2017, and no patients were lost to follow-up. There were missing data for one patient (vital values after 12 hours). The demographics and clinical characteristics at admission of the included patients are presented in Table 1.

**Table 1:** Demographics of and clinical information on the 14 included male patients.

Demographics	Median (range)
Age, years	24 (18–67)
Height, cm	183 (172–198)
Weight, kg	88 (65–110)
Body Mass Index, kg/m^2^	26 (20–32)
**Admission information**	
Blood pressure systolic, mmHg	126 (112–154)
Blood pressure diastolic, mmHg	77 (65–95)
Heart rate, beats per minute	75 (61–107)
Oxygen saturation, %	98 (95–99)
Respiratory frequency, breaths per minute	16 (14–18)
Body temperature, °C	37.5 (36.4–38.1)
White blood cells count, ×10^9^/l	12.5 (6.4–18.3)
C-reactive protein, mg/l	12 (<3–171)
**Surgery**	
Length, hours:minutes	00:47 (00:31–01:53)

**Table 2:** The subjective complaints of the 14 patients evaluated 12 hours, 10 days and 30 days postoperatively after administration of trial treatment. –: none.

Organ system	Complaints after trial treatment (n)		
	12 hours	10 days	30 days
Central nervous system	Dizziness (5)	Dizziness (1)	–
Cardio-pulmonary	Trouble with/discomfort when breathing deeply (4) Coughing (1)	Discomfort when breathing deeply (1)	–
Gastro-intestinal	Bloating (5) No flatus (6) Superficial mild wound pain (2) Mild serous wound secretion (1)	Diarrhoea (3) Dry month (1) Nausea (1) Pain in flank (1)	Intermittent diarrhoea (1)
Urogenital	–		–
Musculoskeletal	–	Gout flare (1)	–
Skin	–	–	–

Numbers in brackets are number of patients.

### Safety

**Figure 2:**
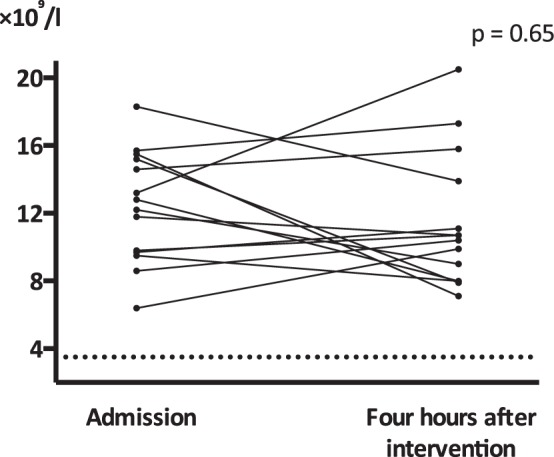
The primary outcome, white blood cell count, at admission and four hours postoperatively (after trial treatment) in 10^9^/l. The toxic limit bellow 3.5 × 10^9^/l is marked with a dashed line. p: p-value, Wilcoxon sign-rank test.

There was no drop of WBC below the predefined toxic range of 3.5 × 10^9^/l. Median WBC four hours after the trial treatment was 10.6 × 10^9^/l (range 7.10–20.5 × 10^9^/l). Furthermore, there was no difference in WBC at admission compared with four hours (±30 minutes) after the trial treatment, Fig. 2 (p = 0.65, Wilcoxon signed-rank test).

### Harms and adverse events

Possible harms were monitored closely during hospitalization for the first 12 hours (±30 minutes) after patients had received the trial treatment. The patients’ subjective complaints during this postoperative period are presented in Table 2. The objective examination was normal in all patients except in one patient, who had a hematoma around the left access port. Possible harms were also assessed at 10 days (±1 day) and 30 days after surgery. Four patients had subjective complaints, see Table 2. One patient experienced dizziness, difficulties breathing deeply, diarrhoea five to six times daily (normal colour, non-blood), and dry mouth at some point during the first 10 days after the surgery. When this patient was contacted 30 days after surgery, he still experienced intermittent diarrhoea and was urged to contact his general practitioner. Two patients also had diarrhoea (normal colour, non-bloody) for the first week after surgery and one patient experienced intense pain of the flank lasting a few seconds 10–15 times a day during the first eight days. Ten days after surgery these three patients had no subjective complaints or objective findings. The objective findings were normal in all patients on the postoperative day 10 days (±1 day) apart from one patient, who had redness and swelling of the foot due to a gout flare, in whom treatment had been started by a general practitioner a few days earlier.

We registered four adverse events in four patients, however, it was impossible to define the relationship to study drugs or to the surgical condition and trial treatment. The three patients with diarrhoea after discharge were regarded as having adverse events. For two of these patients the diarrhoea lasted one week. The third patient had intermittent diarrhoea for a period of at least 30 days. The last adverse event was seen in a patient, who had an episode of dizziness and slight hypotension lasting from 2–12 hours after the trial treatment. Haemoglobin was stable. The patient was treated with Trendelenburg position and intravenous fluids with good effect. The episode did not prolong the patient’s hospital stay. He neither had symptoms after 12 hours nor had complaints after discharge and the end of follow-up 30 days after surgery. There were neither any serious adverse events nor any unexpected serious adverse reactions. There were no infectious complications at the postoperative visit 10 days after surgery or assessed by phone 30 days after surgery at the end of follow-up.

### Biochemical markers

The biochemical markers at admission and four hours (±30 minutes) after the trial treatment are shown in Table 3. Lymphocytes and coagulation factor II, VII, and X decreased significantly and CRP and glucose were significantly increased. The remaining significant changes were still within or nearly within the normal range.

**Table 3:** Overview of the median and range of biochemical markers at admission and four hours postoperatively including normal range and difference (from four hours postoperatively to admission) and statistical analysis.

Biochemical marker	Normal range	Admission	4 hours after trial treatment	Difference	Unadjusted p-value^†^	Adjusted p-value^‡^
**Differential count**						
Basophils, ×10^9^/l	0–0.1	0.05 (0–0.13)	0.02 (0–0.07)	−0.02 (−0.11–0.06)	0.18	0.25
Eosinophils, ×10^9^/l	0.04–0.5	0.16 (0–0.23)	0.14 (0–0.23)	0.0 (−0.19–0.13)	0.59	0.65
Monocytes, ×10^9^/l	0.2–0.76	0.7 (0.13–1.33)	0.54 (0–1.03)	−0.16 (−0.8–0.6)	0.049	0.09
Neutrophils, ×10^9^/l	1.6–5.9	9.44 (3.07–15.95)	8.89 (5.95–18.82)	0.64 (−7.90–8.72)	0.76	0.77
Lymphocytes, ×10^9^/l	1–3.5	1.52 (0.58–3.09)	0.65 (0.39–0.83)	−0.99 (−2.54–0.06)	<0.01	<0.01
Large unstained cells, ×10^9^/l	<0.2	0.11 (0–0.25)	0.09 (0–0.73)	−0.07 (−0.16–0.73)	0.12	0.17
**Kidney function tests**						
Creatinine, µmol/l	60–105	81 (63–96)	87 (74–109)	6 (−5–21)	0.02	0.03
Urea, mmol/l	3.2–8.1	4.9 (3.3–6.8)	4.7 (3.1–6.5)	−0.3 (−1.5–1.3)	0.7	0.73
GFR, ml/minute	>60	114 (81–131)	106 (77–127)	−7 (−23–7)	0.02	0.04
**Liver function tests**						
ALAT, U/l	10–70	40 (20–93)	36 (18–95)	−0.5 (−17–10)	0.40	0.51
Bilirubin, µmol/l	5–25	14 (6–23)	15 (7–33)	4 (−8–11)	0.07	0.11
Albumin, g/l	36–45	45 (41–50)	42 (32–47)	−4 (−18–2)	<0.01	<0.01
INR	<1.2	1.0 (0.9–1.2)	1.1 (1.0–1.4)	0.1 (−0.1–0.0)	<0.01	<0.01
Coagulation factor II, VII and X, %	>63	94 (61–123)	73 (46–92)	−21 (−31–2)	<0.01	<0.01
Thrombocytes, ×10^9^/l	145–390	208 (160–286)	207 (161–287)	−4.5 (−50–21)	0.66	0.71
**Electrolytes**						
Potassium, mmol/l	3.5–4.6	3.9 (3.5–4.3)	4.2 (3.7–5.0)	0.2 (−0.4–1)	0.02	0.03
Sodium, mmol/l	137–144	141 (136–144)	139 (137–143)	−3 (−5–4)	0.01	0.03
**Others**						
CRP, mg/l	<10	12 (<3–171)	41 (11–190)	22 (−19–70)	<0.01	<0.01
Haemoglobin, mmol/l	8.3–10.5	9.5 (8.1–10.6)	9.0 (7.4–9.5)	−0.5 (−2.7–0.1)	<0.01	<0.01
Glucose, mmol/l	4.2–6.3	5.6 (5.1–8.3)	8.1 (6.6–10.5)	2.1 (−1.2–5.4)	<0.01	<0.01
Amylase, U/l	25–120	57 (46–93)	47 (33–78)	−8 (−23–0)	<0.01	<0.01

^†^Wilcoxon sign-rank test, ^‡^false discovery rate^35^, ALAT: alanine transaminase, CRP: C-reactive protein, eGFR: estimated glomerular filtration rate, INR: international normalized ratio.

### Vital signs

The vital signs: systolic and diastolic blood pressure, respiratory frequency, oxygen saturation, and body temperature differed significantly (all p < 0.01) over the whole course of the trial from admission to 10 days after trial treatment, however heart rate was unchanged (p = 0.07).

We investigated the difference in vital signs measured during and without anaesthesia to assess the effect of anaesthesia. Heart rate, respiratory frequency, and oxygen saturation was unchanged for measurement both during and without anaesthesia. The systolic blood pressure differed significantly during anaesthesia (p = 0.02) with a mean decrease of 10–13 mmHg. No difference was found for the systolic measurements without anaesthesia (p = 0.16). The diastolic blood pressure differed significantly both during and without anaesthesia (both p < 0.01). The mean decrease during anaesthesia was 3–5 mmHg. Furthermore, body temperature changed significantly for measurement without anaesthesia (p < 0.01). However, the body temperature was stable during anaesthesia (p = 0.14).

### Length of stay

As a safety precaution the patients had to be admitted for at least 12 hours after the trial treatment was administered. However, one patient left the hospital earlier due to impatience, thus, the median postoperative length of stay was 14 hours (range 5–20 hours).

## Discussion

The combination of fosfomycin, metronidazole, and rhGM-CSF was safe to administer intraperitoneally in patients suffering from uncomplicated acute appendicitis. However, there were some possible harms including subjective complaints of dizziness, lack of flatus, and bloating during the first 12 hours after trial treatment. Four possible adverse events were registered. Three patients presented with diarrhoea after discharge and one patient had a hypotensive episode lasting 10 hours. Some biochemical markers differed from admission to four hours after the trial treatment. However, we could not distinguish due to the trial treatment, infection, anaesthesia, or surgery.

It was safe to administer fosfomycin, metronidazole, and rhGM-CSF in combination. Our primary outcome was to ensure no drop of WBC and no toxic effect four hours after the trial treatment caused by intraperitoneal administration of rhGM-CSF. No leukopenia was detected. WBC count did not differ from admission to four hours after the trial treatment. Leukopenia was chosen as the primary outcome based on previous trials administering rhGM-CSF^10,12,21^. A small phase I trial reported decreased neutrophils and WBC after administering 1 µg/kg per day rhGM-CSF both intravenously and intraperitoneally^10^. The latter resulted in a delay of leukopenia with a maximum decrease 60 minutes after intraperitoneal and 30 minutes after intravenous administration. A similar pattern was seen in a trial administering 0.3–30 µg/kg per day^21^. Only neutrophils decreased with a maximum drop 15–60 minutes after subcutaneous and 3–45 minutes after intravenous administration^21^. The other studies have reported a drop two hours after intravenous administration of 3.2–20 µg/kg per day rhGM-CSF^12^ and 5 minutes after an intravenous bolus of 8 µg/kg per day rhGM-CSF^13^. In the last-mentioned studies, leucocytes, neutrophils, monocytes, and eosinophils decreased; however, there was no drop of lymphocytes^12,13^. Based on these reports, leukopenia could have been present but undetected, because no tests were collected from the beginning of the trial treatment and during the following four hours. However, if leukopenia was present, it would be a transitory phenomenon and therefore no safety issue after intraperitoneal administration. Another explanation could be that rhGM-CSF administered intraperitoneally does not have a systemic effect at a dose of 50 µg (ranging from 0.45–0.77 µg/kg). Only two patients in the first-mentioned trial received rhGM-CSF intraperitoneally and they suffered from intraperitoneal malignancy and had completed chemotherapy^10^.

A possible harm of the trial treatment (or the surgical procedure per se) was diarrhoea after discharge, which was seen in three patients. Diarrhoea is a common harm of antibacterial agents and a common side-effect of intravenously administered metronidazole^22^. However, previous trials, which applied metronidazole intraperitoneally, did not report any adverse events^15,16^. Diarrhoea after treatment with fosfomycin has been reported after both oral^23^ and intravenous administration^24^. Previous trials investigating intraperitoneal administration of fosfomycin lacked statements on adverse events, so we can neither confirm nor dismiss possible harms due to fosfomycin^17,18^. We did not observe any infectious complications after administration of 4 g fosfomycin and 1 g metronidazole intraperitoneally. This is in line with a previous trial that administered 4 g fosfomycin and 0.5 g metronidazole intravenously in patients suffering from uncomplicated appendicitis^25^. Based on this, our preliminary conclusion is that the antimicrobial coverage of fosfomycin and metronidazole administered intraperitoneally is sufficient. This is in line with the results of our *in vitro* study that found a preserved antimicrobial effect of the drugs in combination^20^.

rhGM-CSF have been reported to cause a “first dose effect” defined as subjective complaints and affected vital signs with e.g. hypotension, tachycardia, and hypoxia that appears 15–20 minutes after rhGM-CSF administration and is of short duration^12,21^. We monitored this closely but made no such observations. We did find a decrease in systolic and diastolic blood pressure both over the whole course of the trial and the measurements during anaesthesia shortly after the trial treatment. However, though the decrease was statistically significant, a mean decrease of 10–13 mmHg in systolic and 3–5 mmHg in diastolic blood pressure during anaesthesia is not a clinically important decrease. Furthermore, no tachycardia or hypoxia was observed in this period. Although one patient receiving the trial treatment had a period of slight hypotension, it was not considered to be a “first dose effect” since it lasted 10 hours. Another plausible explanation for the hypotension could be anaesthetics or acute depletion of volume due to prolonged fasting and dehydration.

A standard panel of biochemical markers was taken at admission and four hours after the trial treatment. Some changed significantly between these two measurements. However, most of these had still values within or nearly within the normal range. Hence, we do not consider these of clinical relevance. We saw a decrease in lymphocytes and coagulation factor II, VII, and X as well as an increase in CRP and glucose. Again, numerous explanations could account for these changes. The increase in the infection marker CRP, the decrease in coagulation factors, and the increase in glucose reflected probably pathophysiological changes due to the infection itself or surgery and fasting. Some biochemical markers showed a different change than expected. Lymphocytes fell below the lower normal range in all patients. As previously mentioned, no drop of lymphocytes has previously been reported after intravenous administration^12,13^ nor after subcutaneous administration^26^. Sodium decreased but remained within normal range and potassium increased with the highest value of 5.0 mmol/l being only slightly elevated above the upper normal limit of 4.6 mmol/l. We used fosfomycin disodium, which has a high content of sodium (1.28 g in the administered dose)^27^. Accordingly, previous trials have reported an increase in sodium after intravenous treatment^28,29^. Our combination of drugs was isoosmolar because fosfomycin was dissolved in 500 ml^20^. Furthermore, previous studies have reported hypokalaemia after intravenous administration of fosfomycin^30–32^. We did not find hypokalaemia, however, the previous studies administered a higher total daily dose and/or for a longer period than in our trial.

This phase II clinical trial has several strengths. First, we had conducted *in vitro* investigations prior to the initiation of the trial to ensure a balanced solution and a preserved antibacterial effect^20^. Secondly, harms and adverse events were assessed according to a predefined protocol. We monitored patients closely during the first 12 hours at the hospital. A follow-up with ambulatory visits 10 days postoperatively was conducted and patients were contacted by phone 30 days after surgery. Therefore, no adverse events or harms were overlooked. Ethical reasons made us conduct the trial in otherwise healthy men undergoing laparoscopic appendectomy. Treatment of appendicitis requires an intraperitoneal access and antibacterial agents. It would have been unethical to introduce an intraperitoneal access in healthy volunteers. Furthermore, this resulted in a homogeneous group of participants. The most important limitation is that no control group was included. This was a pilot trial and the first investigation of the trial drugs. Therefore, we did not include a control group as we only aimed to assess the safety and not the efficacy of the intervention compared with a control regimen. The recorded harms could be directly related to the trial treatment, the anaesthesia, or the surgery itself. The trial was conducted in a relatively low number of participants. There is a fine balance between exposing enough participants and minimizing the risk of harms.

In conclusion, intraperitoneal fosfomycin, metronidazole and rhGM-CSF in combination was safe in patients undergoing laparoscopic appendectomy for uncomplicated acute appendicitis. This regimen has few possible harms and adverse events and it is unclear if these were caused by the trial treatment itself or by the infection, anaesthesia, or surgery. These data provide basis for further human studies of this treatment in patients with peritonitis.

## Methods

### Study design and approvals

This prospective phase II clinical trial regarding safety (EudraCT no.: 2015-005772-16) was approved by the Danish Medicines Health Authority (2016022871), the local ethics committee (H-160186070), and the Danish Data Protection Agency (HGH-2016-054) prior to the initiation of the trial. Furthermore, our protocol was registered at clinicaltrials.gov (NCT03046758, 08/02/2017) prior to initiation. The trial was monitored by the Good Clinical Practices (GCP) unit, Copenhagen University Hospital according to Danish and international law.

### Participants

The inclusion criteria were: men ≥18 years old with suspected acute appendicitis and planned for diagnostic laparoscopy and eventual appendectomy, who gave written informed consent after written and verbal information.

Patients were excluded if they fulfilled any of the following exclusion criteria: could not understand, read, or speak Danish; had previous allergic reaction to fosfomycin, metronidazole, or rhGM-CSF; perforated appendicitis (diagnosed either during surgery or at a preoperative CT-scan); diagnostic laparoscopy revealing normal appendix not requiring an appendectomy; other intra-abdominal pathology requiring surgical intervention (diagnosed either during surgery or at a preoperative CT-scan); known renal or hepatic disease or biochemical evidence at the time of admission; known autoimmune disease or other chronic inflammation; known hematologic disease or cancer; previous abdominal surgery (either laparoscopic or open surgery); daily use or use of medication one week prior to or during the trial period apart from painkillers such as paracetamol, ibuprofen, tramadol, and morphine as well as drugs needed for anaesthesia, thrombosis prophylaxis, and nausea; use of other antimicrobial agents than the trial treatment one month before and until 24 hours after the trial treatment; participant in another drug trial one month prior to the date of the surgery, body mass index ≥35 kg/m^2^; or weekly intake of alcohol >14 units, where one unit corresponds to 12 g alcohol.

Potential participants were recruited at the Department of Surgery, Helve Hospital, Denmark. We screened all male patients ≥18 years old that were planned for a diagnostic laparoscopy because of suspected acute appendicitis. Patients, who fulfilled the inclusion criteria and no exclusion criteria, were enrolled. Patients were only included if the operating surgeon or a supervisor visually confirmed the diagnosis of uncomplicated appendicitis during surgery. Only included participants received the trial treatment. If a patient was excluded during surgery, intravenous antibacterial agents were administrated during surgery according to routine clinical practice. The trial personnel informed all enrolled patients whether they had been included in or excluded from the trial at the post-anaesthesia care unit as soon as the effects of the anaesthesia had ended. The indication for diagnostic laparoscopy and the perioperative diagnosis was established by surgeons or their supervisors not involved in the trial.

### Intervention

Laparoscopic appendectomy was performed according to routine clinical practice. When the appendix had been removed from the abdominal cavity and haemostasis had been achieved, the abdominal cavity was irrigated with a minimum of 500 ml of sterile saline. Hereafter, the combination of the trial drugs was instilled in the abdominal cavity (intraperitoneally) in the right fossa through the irrigation/suction device and left there. The trial treatment consisted of a combination of: 4 g fosfomycin (Infectofos, Infectopharm, Germany) diluted in 300 ml of sterile water for injections (Sterilt Vand “SAD”, Amgros I/S, Denmark), 1 g metronidazole (Metronidazol “B. Braun”, B. Braun) corresponding to a volume of 200 ml, and 50 µg molgramostim, rhGM-CSF expressed in *E. coli* bacteria (Repomol, Reponex Pharmaceuticals Aps, Denmark), in a volume of 0.2 ml. The drugs were mixed prior to instillation but administered immediately after to ensure full antimicrobial effect^20^. The instilled volume was 500.2 ml. Our routine antimicrobial treatment for uncomplicated appendicitis consists of a single perioperative dosage of 4/0.5 g piperacillin/tazobactam and 1.5 g metronidazole administered intravenously. However, the study participants did not receive any intravenous or oral antimicrobial treatment, because we aimed to evaluate the safety of the trial treatment, including antibacterial agents with preserved antimicrobial effect^20^.

### Outcomes

**Figure 3:**
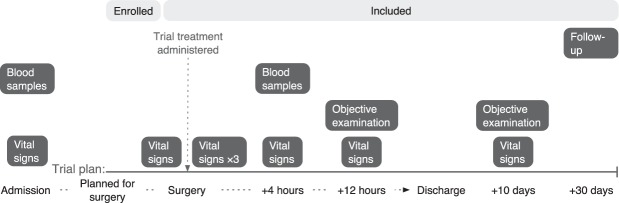
Overview of the trial course of the participants enrolled and included in the trial and the time points for measurement of outcomes. Follow-up consisted of a telephone interview. Vital signs during surgery and anaesthesia were measured prior to and five, ten, and 15 minutes after trial treatment.

An overview of the trial course and the time points for outcome measurements are presented in Fig. 3. The primary outcome was to evaluate safety by measurement WBC. A toxic effect was predefined by a drop of WBC below the lower normal range (3.5 × 10^9^/l) four hours (±30 minutes) after the trial treatment. WBC count four hours (±30 minutes) after the trial treatment were also compared with values at admission.

Secondary outcomes were as follows. Difference in biochemical markers collected at admission (baseline) and four hours (±30 minutes) after the trial treatment: white blood cell differential count (basophils, eosinophils, monocytes, neutrophils, lymphocytes, and large unstained cells); kidney function tests (creatinine, urea, and estimated glomerular filtration rate (eGFR)); liver function tests (alanine transaminase (ALAT), bilirubin, albumin, international normalized ratio (INR), coagulation factor II, VII, and X, and thrombocytes); electrolytes (potassium and sodium), and others (haemoglobin, glucose, and inflammation marker C-reactive protein (CRP)). Change in vital signs (blood pressure, heart rate, respiratory frequency, oxygen saturation (SAT), and body temperature) measured at admission; during anaesthesia: baseline (prior to the trial treatment), five minutes (±five minutes), 10 minutes (±five minutes), and 15 minutes (±five minutes) after the trial treatment; and postoperatively: four hours (±30 minutes), 12 hours (±30 minutes), and 10 days (±1 day) after the trial treatment. We also recorded postoperative length of stay in hours. The primary investigator or trial personnel met with participants and assessed these four hours (±30 minutes), 12 hours (±30 minutes), and 10 days (±1 day) after the trial treatment. The last follow-up was by telephone 30 days after surgery.

Harms, adverse events, serious adverse events, and serious unexpected adverse events were monitored continuously until 30 days after surgery. Serious adverse events were defined as any untoward medical occurrence that at any dose resulted in death, was life-threatening, required inpatient hospitalization or prolongation of existing hospitalization, resulted in persistent or significant disability or incapacity, or resulted in a congenital anomaly or birth defect as defined by ICH-GCP^33^.

A sub-trial regarding the postoperative plasma concentrations and pharmacokinetics of intraperitoneal administration of fosfomycin and metronidazole is currently in writing and will be reported elsewhere.

### Statistics

Data were analysed using SAS Enterprise Guide 7.1 (SAS Institute Inc., USA). Continuous numerical values are reported as mean and standard deviation if normally distributed. If not normally distributed, they are reported as median and range. We tested for normality by visual inspection of histograms and Q-Q plots. Normally distributed continuous data were analysed with a linear mixed model^34^ to account for repeated measurements in each participant where an unstructured covariance was assumed and Goodness of fit was assessed by residual plots). Not normally distributed continuous data were analysed with non-parametric statistics (Wilcoxon signed-rank test). All p-values were adjusted for multiple testing using the Benjamini and Hochberg procedure^35^. An adjusted p-value < 0.05 was considered significant.

### Ethical approval

All procedures performed in studies involving human participants were in accordance with the ethical standards of the institutional and/or national research committee and with the 1964 Helsinki declaration and its later amendments or comparable ethical standards.

### Informed consent

Informed consent was obtained from all individual participants included in the study.

## Supplementary information

Protocol_version1.8_18042016
