# Supplementary material for: Intraperitoneal administration of fosfomycin, metronidazole, and granulocyte-macrophage colony-stimulating factor in patients undergoing appendectomy is safe: a phase II clinical trial
Source: Sci Rep. 2019 Apr 30;9:6727. doi: 10.1038/s41598-019-43151-4 (PMC6491470; doi:10.1038/s41598-019-43151-4)
Supplement: Supplementary file 1 — Protocol_version1.8_18042016 [file 41598_2019_43151_MOESM1_ESM.pdf]

The safety and pharmacokinetics of intraperitoneal administration of granulocyte-macrophage colony-stimulating factor, fosfomycin, and metronidazole in patients undergoing appendectomy for uncomplicated appendicitis

Table of Content

Contact information 5

Sponsor 5

Primary investigator 5

Other investigators 5

Other partners 5

Time frame 6

Introduction 7

Background 8

GM-CSF 8

Fosfomycin 9

Metronidazole 9

The combination of the drugs 10

The dosages of the drugs 11

The formulation of the drugs 12

Intraperitoneal administration of the drugs 13

Trial 13

Main trial: Safety 13

Aim 13

Primary outcome 13

Secondary outcomes 13

Design 14

Trial participants 14

Trial course 15

Withdrawal and dropout criteria 16

Other drugs during the trial 17

Measurements 17

Statistics 18
Sub-trial: Pharmacokinetics 18
Aim 18
Primary outcome 18
Secondary outcomes 19
Design 19
Trial participants 19
Trial course 19
Measurements 20
Statistics 21
Withdrawal and dropout criteria 21
Other drugs during the trial 21
Side effects 22
Side effects of fosfomycin 22
Side effects of metronidazole 23
Side effect of the combination of fosfomycin and metronidazole 23
Side effects of GM-CSF 23
Side effects of the instilled volume 25
Reporting of side effects 25
Pros and cons for the participants of the trial 26
Data monitoring and management 27
Access to relevant documents 27
Quality control and assurance 27
Data management 28
Ethics 28
Ethical considerations 28
Enrolment of participants 28

Finances and insurance 29

Financing 29

Insurance 30

Completion of the trial and publication 30

Publication 30

Summary 31

Supplements 33

References 35

Contact information

Sponsor

Jacob Rosenberg, DMSc, Senior Hospital Physician, Professor, MD

Centre for Perioperative Optimisation (CPO), Department of Surgery, Herlev Hospital, University of Copenhagen, Herlev Ringvej 75, DK-2730 Herlev

Phone: +45 26 23 73 23

Email: jacob.rosenberg@regionh.dk

Primary investigator

Siv Fonnes, MD, PhD student

CPO, Department of Surgery, Herlev Hospital, University of Copenhagen, Herlev Ringvej 75, DK-2730 Herlev

Phone: +45 23 21 65 34

E-mail: siv.fonnes@regionh.dk

Other investigators

Magnus Arpi, Senior Hospital Physician, MD

Department of Clinical Microbiology, Herlev Hospital, University of Copenhagen, Herlev Ringvej 75, DK-2730 Herlev

Phone: +45 38 68 38 83

E-mail: rolf.magnus.arpi@regionh.dk

Barbara Holzknecht, PhD, Staff Specialist, MD

Department of Clinical Microbiology, Herlev Hospital, University of Copenhagen, Herlev Ringvej 75, DK-2730 Herlev

Phone: +45 38 68 38 50

E-mail: barbara.juliane.holzknecht@regionh.dk

Other partners

Johan Juhl Weisser, PhD student

Faculty for Pharmacy, University of Copenhagen

Universitetsparken 2, DK-2100 København Ø

E-mail: johan.weisser@sund.ku.dk

Dorte Kromann Krydsfeldt
Region Hovedstadens Apotek
Marielundvej 25, 2730 Herlev
Phone: +45 44 57 77 07
E-mail: dorte.kromann@regionh.dk

This trial will be conducted in compliance with the present trial protocol, ICH-GCP guidelines and the applicable regulatory requirements. The custom procedures for both quality control systems and quality assurance will be followed according to ICH-GCP guidelines and the investigator and sponsor assure direct access to all trial related sites, source data/documents, and reports for the purpose of monitoring, auditing, and inspection by regulatory authorities.

Time frame

The trial will start shortly after approval from the Danish Medicines Authority, the local Ethics Committee and the Danish Data Protection Agency. Approximately 20 patients are treated for uncomplicated appendicitis at Department of Surgery, Herlev Hospital, per month. Altogether, 14 patients are needed to complete the trial. We estimate that the inclusion of patients will take two to three months. Because of 30 days follow-up, the expected trial period is four months.

Date: 18.04.2016

Jacob Rosenberg
Professor, overlæge, dr.med.
Gastroenheden - Kirurgisk Sektion D113
Herlev Hospital
Herlev Ringvej 75 - 2730 Herlev
Tlf. 3868 9504 - Mobil: 2623 7323
e-mail: jacob.rosenberg@regionh.dk

Signature:

Jacob Rosenberg

Introduction

Peritonitis is an inflammation of the peritoneum, the epithelium that lines the abdominal cavity.[1] The inflammation can occur either locally or diffusely in the abdomen. The severity of the disease is closely related to the extent of the inflammation. A local inflammation as seen in uncomplicated appendicitis is an acute, reversible and less severe disease than diffuse peritonitis secondary to a perforation of the colon with faecal contamination throughout the abdomen.[2] In the latter case, peritonitis can have long-term adverse consequences for the patient even with the best current treatment options.

Current practice for both local and diffuse peritonitis is surgical intervention (i.e. source control) supplemented with the systemic (intravenous) administration of antibiotics. We wish to conduct a trial to investigate a new combination and new route of administration of drugs to treat peritonitis in combination with the current standard surgical intervention. The drugs are administered intraperitoneally, i.e. locally at the site of the disease. Thereby the highest concentration of the drugs is achieved at the site of the infection: in the abdominal cavity. Therefore, we expect that the trial drugs will act more efficiently and result in fewer side effects. Further, the treatment consists of an alliance of two types of drugs. These drugs are antibiotics and a drug that improves the patients' own immune defence. This combination attacks peritonitis from more angles that the existing treatment. The antibiotics fosfomycin and metronidazole have direct antimicrobial actions. The patient's own local immune defence is strengthened by granulocyte-macrophage colony-stimulating factor (GM-CSF), which improves the function of macrophages at the site. Thereby, the bacteria can be eliminated more efficiently.

We wish to improve the treatment of patients suffering from peritonitis. This pilot trial is conducted in the healthiest group of patients suffering from local peritonitis: patients with uncomplicated appendicitis. These patients have few complications and are often ready for discharge some hours after surgery at which the appendix is removed. The intraperitoneal administration of drugs requires access to the abdominal cavity, which can only be gained by an invasive

procedure. Therefore, healthy volunteers are not suitable for this pilot trial as this kind of invasive procedure would not be justifiable.

Background

GM-CSF

GM-CSF is a glycoprotein[3,4] cytokine, which is produced by cells of the immune system such as macrophages, monocytes and T lymphocytes, as well as by fibroblasts, endothelial cells[5] and many types of epithelial cell. The cytokine binds to its receptor on the cell membrane and activates a signalling pathway inside the cell.[6] Thereby, GM-CSF promotes the growth, differentiation and maturation of macrophages and neutrophil granulocytes and stimulate their activities.[3,5,7]

Recombinant human GM-CSF has been synthesized in various host cells, such as *Escherichia coli* (*E. coli*),[8] yeast,[9] or mammalian cells.[10] The following sections concern the use of the non-glycosylated recombinant human GM-CSF produced by *E. coli*: molgramostim.

Recombinant GM-CSF has been administered by different routes: subcutaneously, intravenously, and intraperitoneally. GM-CSF administered intraperitoneally improved the local immune response in patients undergoing dialysis[11] and suffering from advanced intra-abdominal cancer.[12] Here, an intraperitoneal sample from one patient confirmed an increase in white blood cells and neutrophil granulocytes inside the abdominal cavity.[12] Another study also found that the numbers of macrophages, lymphocytes, and neutrophil, eosinophil and basophil granulocytes in peritoneal effluent showed a significant increase as a response to intraperitoneal administration of GM-CSF.[11] Further, the phagocytic activity of peritoneal macrophages improved.

The subcutaneous administration route of GM-CSF has been studied in a randomised controlled trial (RCT) in patients treated for abdominal sepsis.[13] Subcutaneously administered GM-CSF resulted in faster improvement, development of fewer infectious complications, and fewer reoperations. Further, GM-CSF was intravenously administered in an RCT in patients with severe sepsis and respiratory dysfunction admitted to the intensive care unit.[14] GM-CSF was

associated with improved oxygenation and increased numbers of neutrophil granulocytes in the blood.

Fosfomycin

Fosfomycin is an organic phosphonate[15,16] antibiotic of low molecular weight and negligible protein binding, which shows a good tissue distribution.[17,18] The half-life in blood of fosfomycin is approximately 2 hours.[19,20] It is excreted unchanged into the urine by glomerular filtration.[21] It enters the bacteria and inhibits the bacterial cell wall synthesis through an irreversible inhibition of the enzyme enolpyruvyl transferase.[16] This results in a bactericidal activity on bacteria in the growth phase.[22] Fosfomycin has a broad spectrum of activity against both Gram-positive and Gram-negative aerobic bacteria.[15] Intravenous fosfomycin has been used for abdominal- and other severe infections and is increasingly used to treat infections with multi-drug-resistant bacteria. Its antibacterial spectrum includes *E. coli* and other *Enterobacteriaceae*, including those producing extended spectrum beta-lactamase (ESBL).[23,24] Further, it shows good activity against *Staphylococcus aureus*, including methicillin-resistant *Staphylococcus aureus* (MRSA),[23,25] and moderate activity against enterococci, especially *Enterococcus faecalis*, even when these are vancomycin resistant (VRE).[26] The oral formulation, fosfomycin trometamol, is used routinely to treat uncomplicated urinary tract infections in southern Europe.[27]

Fosfomycin has been administered intraperitoneally in patients undergoing dialysis.[28,29] Both studies were pharmacological studies in patients without signs of peritonitis and suggested bidirectional exchange of fosfomycin from the blood to the abdominal cavity and vice versa.[28,29] One of the studies found a better systemic absorption of intraperitoneally administered fosfomycin than penetration of intravenously administered fosfomycin into the peritoneum.[28] This indicates that intraperitoneal administration of fosfomycin could have an advantage over intravenous administration.

Metronidazole

Metronidazole is a nitroimidazole antibiotic, which has a bactericidal action on anaerobic bacteria and also kills certain protozoa, e.g. amoebae.[30] The half-life of

metronidazole in the circulation is 6-8 hours.[31] It is metabolised in the liver to give rise to five different metabolites, which have varying levels of antimicrobial activity that are lower than that of metronidazole. Metronidazole and its metabolites are mostly excreted into the urine. It has been estimated that less than 20% of metronidazole is bound to plasma proteins.[32,33] The mechanism of action is not fully understood. However, cytotoxic intermediate metabolites interact with bacterial DNA and result in destabilisation.[31]

Because of its anaerobic coverage, it plays a prominent role in both the prophylaxis and treatment of intra-abdominal infections.[31] Metronidazole is usually administered intravenously, orally or rectally. However, vaginal and topical routes of administration routes also exist.

Metronidazole has been given intra-abdominally. One study examined 500 mg metronidazole and 500 mg cephradine installed in the peritoneal cavity and wound in 46 patients undergoing laparotomy where faecal or purulent peritonitis or faecal contamination of the wound was present.[34] The antimicrobial agents were left for 1½ hours before they were drained through draining tubes. Another study applied 500 mg or 1 g metronidazole to the abdominal cavity, which was removed through drains after 2 hours, in 182 patients with faecal or purulent peritonitis or faecal soiling of the abdominal cavity.[35] These patients also received intravenous administration of antimicrobial agents. Finally, a study, which divided a total of 100 patients with complicated appendicitis into intervention and control groups, administered both intravenous antimicrobial agents and a local installation of 500 mg of metronidazole and 1 g of cephazolin in the peritoneal cavity and wound layers in the intervention group.[36] The control group only received the intravenously administered antimicrobial agents and had more early complications than the intervention group. None of the abovementioned studies made pharmacokinetic investigations.

The combination of the drugs

The combination of GM-CSF, fosfomycin, and metronidazole has to our knowledge not been given before. However, fosfomycin and metronidazole has previously been co-administered intravenously to patients undergoing

abdominal surgery.[37,38] Both for elective[38] and emergency[37] surgery the combination was effective. It was more effective than control treatment with metronidazole and gentamicin in a study of emergency surgery.[37] Therefore, we expect the combination of these antibiotics intraperitoneally to be a reasonable alternative treatment to the current combination of intravenously administered beta-lactam antibiotics and metronidazole. A previous RCT treated abdominal sepsis with antimicrobial agents intravenously together with subcutaneously administered GM-CSF.[13] These patients improved faster, developed less infectious complications, and needed fewer reoperations. Therefore, we expect the stimulation of the patients' local immune defence by GM-CSF to have an additive or synergic effect with respect to antimicrobial therapy alone.

The dosages of the drugs

We have chosen doses of 50 μg of GM-CSF (molgramostim) 4 g of fosfomycin, and 1 g of metronidazole. The chosen doses are based on doses from previous studies and clinical practice.

Intraperitoneal doses of GM-CSF of 68 μg/day[11] were administered in patients undergoing peritoneal dialysis and intraperitoneal doses of 1, 2, 4, or 8 μg/kg/day were administered to patients with advanced intraperitoneal malignancy.[12] Pharmacokinetic studies have administered escalating intravenous doses of GM-CSF of 1, 2, 4, or 8 μg/kg/day to patients with advanced intraperitoneal malignancy,[12] 0.3, 1, 3, 10, 15, and 20 μg/kg/day to patients with advanced malignancy and/or neutropenia,[39] and 3 μg/kg or 5.5 μg/kg to patients with malignant lymphomas.[40] An RCT administered an intravenous dose of 3 μg/kg/day to patients requiring admission to the intensive care unit.[14] Pharmacokinetic studies administered subcutaneous doses of GM-CSF of 5.5 μg/kg to patients with malignant lymphomas,[40] and 0.3, 1, 3, 10, 15, 20, and 30 μg/kg/day to patients with advanced malignancy.[41] In an RCT patients with abdominal sepsis received subcutaneous doses of 3 μg/kg/day.[13]

Our chosen dose of 50 μg of GM-CSF is low compared to these studies. This conservative dosing regimen decreases the risk of adverse effects but is expected to be high enough to have a local intra-abdominal effect.

The two previous pharmacokinetic studies on fosfomycin administered intraperitoneally used doses of 1 g[29] and 4 g.[28] The previous studies where intravenous fosfomycin was administered during emergency abdominal surgeries used a dose of 4 g.[37] For the treatment of severe infections the recommended intravenous dosage is 12-24 g daily divided into 3-4 doses.[42,43] Our chosen dose of 4 g is at the same level as previous studies and current clinical practice.

It is clinical practice to give a systemic, intravenous infusion of metronidazole in combination with other antimicrobial agents during emergency surgical procedures or when contamination of the abdominal cavity is expected. The standard hospital guideline for a single dose is 1 g of metronidazole, whereas continuous treatment consists of 1-1.5 g of metronidazole daily divided into 2-3 doses. The previous studies with intra-abdominal administration used 500 mg[34–36] to 1 g.[35] We have chosen a dose of 1 g of metronidazole, which is within the range of the above-mentioned doses.

The combined dosages will only be administered once – during surgery – which is similar to the current clinical practice for the intravenous administration of antibiotics during surgery for uncomplicated appendicitis.

The formulation of the drugs

The formulation of fosfomycin Fomicyt/Infectofos manufactured by Infectopharm is used in this trial and approved for clinical use in Germany. The 4 g of fosfomycin will be diluted in 300 ml of sterile water.

Metronidazole is available in our department as 100 ml *Metronidazol "B. Braun"*, which has a concentration of 5 mg/ml. We will use 200 ml of metronidazole.

The GM-CSF used will be Repomol (molgramostim) from Reponex Pharmaceuticals A/S, which consists of recombinant human GM-CSF expressed in *E. coli* bacteria. We will use a dose of 50 μg in 0.2 ml of solution (water for injections).

The total volume administered intraperitoneally will be 500 ml.

Intraperitoneal administration of the drugs

Intraperitoneal administration of antimicrobial agents has been recommended in the treatment of peritonitis associated with peritoneal dialysis[44–47] and is accepted clinical practice at our hospital.

The drugs will be administered through an already established access to the abdominal cavity. During a laparoscopic appendectomy, three small accesses are made. These are used for a camera and instruments. One of these instruments includes a suction tube used both for suction of excess fluid and irrigation with saline. When the removal of the appendix is completed and haemostasis is secured, the abdominal cavity will be irrigated with a minimum of 500 ml of saline. Hereafter, the drugs will be administered into the intraperitoneal space through the suction tube.

Trial

The trial will consist of a main trial of safety and a sub-trial on pharmacokinetics. These involve and will be recruited from the same trial population of patients. The main trial on safety and the sub-trial on pharmacokinetics will be described independently in the following sections.

Main trial: Safety

Aim

We wish to evaluate the safety of the intraperitoneal administration of the combination of fosfomycin, metronidazole, and GM-CSF.

Primary outcome

The safety of intraperitoneal administration is evaluated through the white blood cell counts 4 hours postoperatively. A toxic effect is defined by a drop below the lower reference range.

Secondary outcomes

Repeated biochemical markers (including a white blood cell differential count, inflammation marker C-reactive protein (CRP), kidney function tests, liver

function tests, and electrolytes), vital signs (blood pressure, pulse, frequency of respiration, peripheral saturation (SAT), and temperature,), length of stay, side effects, and adverse events until 30 days after surgery.

Design

An open-label, phase II, pilot study of 14 participants.

Trial participants

Inclusion criteria:

- Men ≥18 years old
- Suspicion of acute appendicitis and planned for diagnostic laparoscopy and eventual appendectomy
- Written informed consent after written and verbal information

Exclusion criteria:

- Cannot understand, read or speak Danish
- Previous allergic reaction to fosfomycin, metronidazole, or GM-CSF
- Perforated appendicitis (diagnosed either during surgery or at a preoperative computer tomography (CT) scan)
- Diagnostic laparoscopy revealing normal appendix not requiring an appendectomy
- Other intra-abdominal pathology requiring surgical intervention (diagnosed either during surgery or at a preoperative CT-scan)
- Known renal or hepatic disease or biochemical evidence at the time of admission
- Known autoimmune disease or other chronic inflammation
- Known hematologic disease or cancer
- Previous abdominal surgery (either laparoscopic or open surgery)
- Daily use or use of medication one week prior to or during the trial period apart from painkillers such as paracetamol, ibuprofen, tramadol, and morphine as well as drugs needed for anaesthesia, thrombosis prophylaxis, and nausea. Limitations for antibiotics are defined below

- Use of other antimicrobial agents than the trial treatment one month before until 24 hours after the trial treatment
- Participant in another drug trial one month prior to the date of the surgery
- Body mass index ≥35 kg/m^2
- Weekly intake of alcohol >14 units, where one unit corresponds to 12 g alcohol

Trial course

The trial course is outlined in Figure 1. Patients are admitted to the Department of Surgery, Herlev Hospital, with suspected acute appendicitis and a general panel of blood samples are collected. A surgeon from the department will either plan for an acute surgery or keep patients for further observation. The primary investigator of the trial approaches these otherwise healthy patients to give information about the trial. Thereby we ensure the patient has plenty of time for consideration in this setting where an acute disease is involved. Patients, who fulfil the inclusion criteria and present none of the exclusion criteria, apart from those criteria that can only be clarified at surgery, are enrolled in the trial after informed consent. Baseline characteristics of the enrolled patients will be noted in the case report form (CRF). A note of the patient's enrolment in the trial will be added to the patient's medical record, so the standard intravenous antibiotics are not administered. The patients are included in the trial during the surgery when the operating surgeon or a supervisor visually confirms the diagnosis of uncomplicated appendicitis. Patients that fulfil any exclusion criteria are excluded from the trial and will receive treatment and antibiotics according to standard clinical practice. Included patients will have their appendixes removed laparoscopically according to standard clinical practice. When the appendix has been removed from the abdominal cavity through an Endobag and haemostasis has been secured, a minimum of 500 ml of saline will be used for irrigation of the abdominal cavity. Hereafter the trial treatment will be instilled into the abdominal cavity under the supervision of the trial investigator to ensure full compliance. The patients will have vital signs measured at baseline, 5 minutes, 10 minutes, 15 minutes, 4 hours and 12 hours after the trial treatment during

their hospital admission. These values will be noted in the CRF. Participants will be peri- and postoperatively monitored according to standard practice. Postoperatively, the general panel of blood samples will be repeated 4 hours after the trial treatment, which differs from standard clinical practice. Side effects are evaluated through the following procedures. The primary investigator will perform an objective examination of the participant and ask if any changes have risen 12 hours after the trial treatment has been administered. This will be noted in the CRF. When the patient has a "Postanesthetic Recovery Score for Patients Having Anesthesia on Ambulatory Basis" (PARSAP)[48] ≥18 and is informed about the procedure the patient will be discharged according to clinical practice. PARSAP is found in Supplements, Table 2.

Ten days after the trial treatment has been administered side effects will be evaluated again. The primary investigator will meet with the participant to perform an objective examination and ask if any changes have risen since the surgery. This will be noted in the CRF.

A follow-up of the participants will be performed 30 days after the surgery through medical records and contact to the participant by telephone, which will be noted in the CRF.

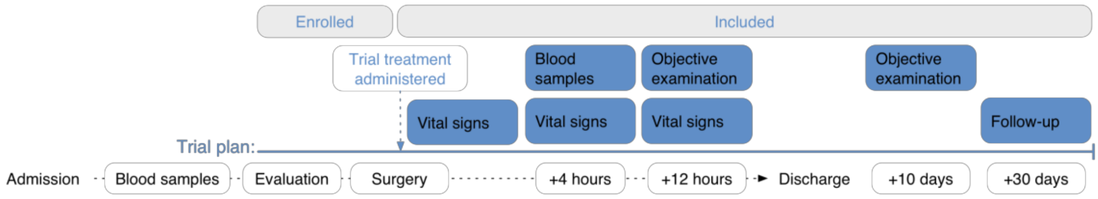

Figure 1. The trial course for participants in the main trial: Safety. An outline (black text) of the normal course of admission for appendicitis is seen at the bottom. The timespan for enrolment and inclusion are marked in grey boxes. All investigations related to the trial are shown in blue text and in the blue boxes. These include blood samples, vital signs (pulse, blood pressure, temperature, frequency of respiration, and SAT), and objective examinations combined with questions about any changes to evaluate side effects. Finally, a follow-up will be conducted 30 days after the surgery. The measurement of vital signs during the surgery will be performed at baseline, 5, 10 and 15 minutes after the trial treatment has been administered.

Withdrawal and dropout criteria

The participants can withdraw their consent at any point during the trial. If the participant becomes unstable during the surgery, the primary investigator can exclude the participant from the trial for the safety of the participant. This will be effectuated in case of:

- Severe intra-abdominal bleeding
- Allergic reaction to general anaesthesia
- Other severe complications during surgery

A participant has concluded the trial when the 30-day postoperative follow-up has been conducted.

The trial will be stopped if severe adverse effects or severe complications, which are not expected, arise from the trial treatment. This will be decided by the sponsor.

Other drugs during the trial

The participants can receive any pain medication (paracetamol, ibuprofen, tramadol, or morphine) necessary before or after the surgery. Further, the patients can receive intravenous fluids without restrictions and the necessary thrombosis prophylaxis. In addition, medication necessary for anaesthesia and nausea can be administered without restrictions.

Measurements

Primary outcome:

- Preoperatively, white blood cell counts at admission (baseline) compared with white blood cell counts 4 hours ± 30 minutes postoperatively are analysed by Department of Clinical Biochemistry, Herlev Hospital according to their usual standards. The blood samples are destroyed after analysis. A toxic effect defined by a drop below the reference range.

Secondary outcomes:

- Preoperative biochemical markers are analysed at admission (baseline) and 4 hours ± 30 minutes postoperatively by Department of Clinical Biochemistry, Herlev Hospital according to their usual standards. These include a standard panel of blood samples, which are seen in Supplements, Table 3. The blood samples are destroyed after analysis. This requires a total of 20.5 ml blood.
- Vital signs (pulse, blood pressure, temperature, frequency of respiration, and SAT) are measured perioperatively (baseline, 5 minutes, 10 minutes

and 15 minutes after the trial treatment has been administered) and postoperatively (4 and 12 hours ± 30 minutes after the trial treatment has been administered).

- Length of stay in hours postoperatively.
- Side effects: evaluated through an objective examination and questions about changes 12 hours ± 30 minutes and 10 days postoperatively ± 1 day.
- Adverse events: registered from the surgery until 30 days postoperatively through medical records and contact with the participant by telephone.

Statistics

The number of patients needed for the trial is not based on a power calculation. This trial is a pilot observational safety trial of the intraperitoneal administration of fosfomycin, metronidazole, and GM-CSF. We have decided that 14 patients are needed to collect a minimum of information about the safety of the intraperitoneal treatment.

Continuous numerical data will be reported as mean and standard deviation if normally distributed. If not normally distributed, continuous numerical data will be reported as median and percentiles. We will analyse data with both parametric and non-parametric statistics depending on the distribution of the data. A p-value <0.05 is considered significant.

Sub-trial: Pharmacokinetics

Aim

We wish to investigate the plasma concentrations of fosfomycin and metronidazole after intraperitoneal administration in patients diagnosed with uncomplicated appendicitis.

Primary outcome

The plasma concentrations of fosfomycin over time are measured with high-performance liquid chromatography mass spectrometry (HPLC-MS).

Secondary outcomes

The plasma concentrations of metronidazole over time are measured with HPLC-MS and microbiological investigations of specimens from appendices and/or abdominal fluid removed during surgery.

Design

An open-label, phase II, pharmacokinetic pilot study of 8 participants.

Trial participants

The inclusion and exclusion criteria are similar to the criteria of the main trial: Safety and are seen in the section Trial participants.

Trial course

The trial course is outlined in Figure 2. We expect that eight of the participants from the main trial on safety also will participate in this sub-trial on pharmacokinetics. The trial course is similar to the course described for the main trial: Safety with the addition of the following. If excess fluid is discovered in the abdomen at the beginning of the surgery, this will be collected for microbiological investigations. When the appendix has been removed from the abdominal cavity through an Endobag, a swab from the appendix will be collected for microbiological investigations before it is sent for pathological evaluation. A baseline blood sample is collected from a large peripheral vein during the surgery immediately before the trial treatment is given. Blood samples will be collected at ½, 1, 2, 4, 8, 12, and 24 hours after the trial treatment from large peripheral veins or through an intravenous access. The blood sample at ½, 1, and 2 hours will be collected with a leeway of ±15 minutes. The blood sample at 4, 8, and 12 hours will be collected with a leeway of ±30 minutes. The blood sample at 24 hours after surgery will be collected with a leeway of ±4 hours. The collection of blood samples will be noted in the CRF.

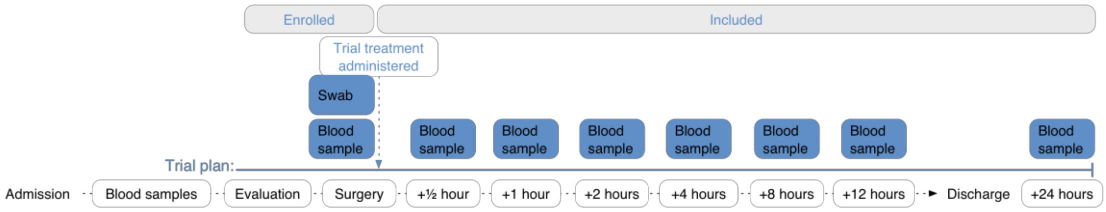

Figure 2. The trial course for participants in the sub trial: Pharmacokinetics. An outline (black text) of the normal course of admission for appendicitis is seen at the bottom. The timespan for enrolment and inclusion are marked in grey boxes. All investigations related to the trial are shown in blue text and in the blue boxes. These include repeated blood samples and a swab from the removed appendix and/or excess fluid in the abdominal cavity.

Measurements

Primary outcome:

- Plasma concentrations of fosfomycin over time measured by HPLC-MS will be analysed at Faculty for Pharmacy, University of Copenhagen. 4 ml of blood will be collected per sample. A research biobank will be established for the collected samples, which will be frozen and stored at -80°C for one year. Thereafter the plasma samples are destroyed. However, the analysed samples without plasma (and thereby without human residues) are stored until the study has been published.

Secondary outcomes:

- Plasma concentrations of metronidazole over time measured by HPLC-MS will be analysed at Faculty for Pharmacy, University of Copenhagen. 4 ml of blood will be collected per sample. A research biobank will be established for the collected samples, which will be frozen and stored at -80°C for one year. Thereafter the plasma samples are destroyed. However, the analysed samples without plasma (and hence without human residues) are stored until the study has been published.
- Microbiological flora and susceptibility: The specimens collected during the surgery from the abdominal excess fluid and/or swab from the appendices are immediately sent to the Department of Clinical Microbiology, Herlev Hospital, where they are investigated. Hereafter, the collected human material is destroyed. However, the cultured bacteria (without human residues) will be frozen at -80°C and stored for 3 years.

Statistics

The number of patients needed for the trial is not based on a power calculation. This trial is a pilot study of the pharmacokinetics of intraperitoneal administration of fosfomycin and metronidazole. We have decided that 8 patients are needed to collect a minimum of information of how the plasma concentrations develop over time.

The continuous numerical data will be reported as mean and standard deviation if normally distributed. If not normally distributed, continuous numerical data will be reported as median and percentiles. Data from HPLC-MS analysis will be used to calculate e.g. the area under the curve (AUC), the maximum concentration observed (C_{max}), the time point for the maximal concentration (T_{max}), and the terminal phase half-life ($T_{½}$).

Withdrawal and dropout criteria

The participants can withdraw their consent at any point during the trial. If the participant becomes unstable during the surgery, the primary investigator can exclude the participant from the trial for the safety of the participant. This will be effectuated in case of:

- Severe intra-abdominal bleeding
- Allergic reaction to general anaesthesia
- Other severe complications during surgery

A participant has concluded the sub-trial after the last blood sample has been collected 24 hours ±4 hours after the trial treatment.

Other drugs during the trial

The patients can receive any analgesic medication necessary before or after the surgery. Further, patients can receive intravenous fluids without restrictions. The medication necessary for anaesthesia, thrombosis prophylaxis, and the treatment of nausea can be administered without restrictions. These will be noted in the CRF.

Side effects

In the following sections the side effects of each drug is described. The side effects of fosfomycin and metronidazole are based on the literature and their respective summary of product characteristics. The summary of product characteristics for fosfomycin and metronidazole will be the reference document for side effects and adverse events.[30,49] The side effects of GM-CSF are based on the available literature. The reference document for side effects and adverse events of GM-CSF will be the Investigators Brochure. The most common side effects are listed in Table 1 and are experienced by 1-10% of patients receiving the drugs.

Type of side effect	Fosfomycin	Metronidazole	GM-CSF
**Gastrointestinal**	Abdominal pain<br>Vomiting	Dry mouth<br>Metal taste<br>Nausea<br>Oral mucositis<br>Stomatitis	Diarrhoea<br>Nausea<br>Vomiting
**Skin**	Injection site phlebitis		Injection site reaction<br>Rash
**Other**			Anorexia<br>Asthenia<br>Dyspnoea<br>Fatigue<br>Fever<br>Musculoskeletal pain<br>Rigors

Table 1. The most common side effect of fosfomycin, metronidazole, and granulocyte-macrophage colony-stimulating factor (GM-CSF).

Side effects of fosfomycin

Fosfomycin is generally well tolerated. A surveillance study of more than 35,000 patients receiving orally administered fosfomycin reported side effects in less than 3.5% of the patients.[50] The most common side effects were gastrointestinal disturbances such as vomiting and abdominal pain,[49] which are common side effects of many antimicrobial agents. Further, after intravenous administration of doses of 4 g fosfomycin 4 times a day in 11 critically ill patients, no adverse events was experienced.[51] Diarrhoea was reported in one child after intravenous doses of 200 mg/kg fosfomycin were administered in 23 children suffering from osteomyelitis.[52] Other studies reported high sodium, which is the dose limiting factor, and more seldom: allergic reactions, headache, rhinitis, vaginitis, and

phlebitis.[15,53,54] In pharmacokinetic studies where fosfomycin was administered intraperitoneally adverse events were not reported.[28,29]

Side effects of metronidazole

Metronidazole is routinely administered intravenously to treat peritonitis e.g. after perforation of the appendix. It is generally well tolerated. The known side effects of intravenous administration include metal taste, nausea, oral mucositis, stomatitis, and dry mouth.[30] Some patients who consume alcohol together with metronidazole experience a disulfiram-like (Antabuse-like) reaction. Further, the urine can be coloured deeply red.

Two studies reported no adverse reactions after a single abdominal installation of metronidazole (500 mg in 100 ml fluid) in the peritoneum and wound during closure after open surgery for complicated appendicitis[36] or laparotomy.[34]

Side effect of the combination of fosfomycin and metronidazole

The combination of fosfomycin and metronidazole has been administered intravenously in patients undergoing either elective[38,55] or emergency colorectal surgery.[37] Only few side effects were reported in these studies and included: gastrointestinal side effects such as nausea and vomiting and skin reactions (urticaria, thrombophlebitis, purpura, and skin rash).[37,38,55]

Side effects of GM-CSF

GM-CSF has overall been reported well tolerated regardless of route of administration.[12,14,39,41] GM-CSF administered intravenously in an RCT in patients requiring admission to the intensive care unit reported reversible oliguria as the only side effect.[14] A pharmacokinetic study of escalating doses (0.3, 1, 3, 10, 15, and 20 μg/kg/day) in patients with advanced malignancy and/or neutropenia reported the following most common adverse clinical effects: 71% bone pain, 62% lethargy/malaise, 38% rash and/or pruritus, 38% first dose reaction, 29% fever, 24% fever and rigors, 14% weight dose gain, 10% marginal keratitis, 10% nasal congestion, and 10% pericardial pain.[39] Adverse effects such as fever, pericarditis, and liver abnormalities were not seen in

patients receiving <10 μg/kg.[56] Also transient leukopenia was seen after intravenous administration.[39] The first dose reaction is characterised by either symptomatic hypotension, hypoxia or both and at least two other common symptoms (flushing sensation, muscular or bone aches, throbbing lumbar back pain, dyspnoea, nausea and vomiting, rigor or fever, and involuntary leg spasms).[57] This happened within 60 ± 40 minutes after intravenous injection or infusion. Subcutaneous administration in patients with sepsis resulted in no side effects.[58] However, the first dose reaction was associated with high serum concentrations and an intravenous administration route.[56] Another pharmacokinetic study reported bone pain, chills/rigors, dyspnoea, fever, serositis and thrombosis after intravenous administration of escalating doses of GM-CSF of 1, 2, 4, or 8 μg/kg/day.[12] One pharmacokinetic study did not report on adverse effects.[40]

An RCT reported the following adverse reactions after a dose of 3 μg/kg/day GM- CSF administered subcutaneously to patients with abdominal sepsis: thrombocytopenia, generalised rash, nausea, deep vein thrombosis, and superficial phlebitis.[13] A pharmacokinetic dose escalating study reported the following adverse effects: fever, bone pain, arthralgia/myalgia, skin reaction, increased liver enzymes, thrombocytopenia and pericarditis after subcutaneously administered doses of 0.3, 1, 3, 10, 15, 20, or 30 μg/kg/day to patients with advanced malignancy.[41] Pericarditis occurred only after high doses.[41,56] The transient leukopenia seen after intravenous administration[39] arose later after subcutaneous administration.[56] One pharmacokinetic study did not report on adverse effects.[40]

Only a transitory flu-like syndrome was reported after doses of 68 μg/day GM-CSF administered intraperitoneally in patients undergoing peritoneal dialysis.[11] A pharmacokinetic study reported fever and serositis after intraperitoneal administration of escalating doses of 1, 2, 4, or 8 μg/kg/day in patients with advanced intraperitoneal malignancy.[12] This study, which also administered GM-CSF intravenously, noted that first dose reaction was seen only after intravenous and not intraperitoneal administration.

We expect fewer and less serious side effects of GM-CSF than described in the above studies because of the intraperitoneal administration route and the low dose that has been chosen.

Side effects of the instilled volume

After the intraperitoneal administration of drugs, a volume of 500 ml will remain as an instillation in the abdominal cavity, which will then be gradually absorbed. In a recent RCT, 500 ml of saline were instilled as an intervention after laparoscopic cholecystectomy to investigate effects on pain, recovery and quality of life.[59] The control group did not receive any fluid instillation. There was no difference in pain, recovery, or quality of life between the two groups. The only significant outcome was increased nausea one hour after surgery in the intervention group. However, there was no difference at the subsequent time points at four hours, one day, and seven days postoperatively. No other adverse effects were reported. Therefore we expect the planned volume of 500 ml to be without side effects for the patients.

Reporting of side effects

The participants will be asked if they have experienced any changes both 12 hours and 10 days after the surgery in combination with an objective examination. This will be documented in the CRF. Further, the patient will be asked if they have experienced any adverse events defined as any unfavourable and unintended sign, symptom, or disease associated with the intraperitoneal treatment, whether or not related to that treatment. A follow-up is conducted 30 days after surgery. This will also be documented in the CRF and will be included in the final report that is sent to the Danish Medicines Authority after the trial.

Serious adverse events (SAE) or serious adverse drug reactions are defined as any untoward medical occurrence that at any dose: results in death, is life-threatening, requires inpatient hospitalization or prolongation of existing hospitalization, results in persistent or significant disability or incapacity, or results in a congenital anomaly or birth defect as defined by ICH-GCP. SAE or serious adverse drug reactions will be reported to the sponsor immediately by the primary investigator.

Unexpected serious adverse reactions (SUSAR) are defined as a SAE or serious adverse drug reactions for which there is a probable causal connection and which is unexpected from the information given in the reference documents (fosfomycin and metronidazole: summary of product characteristics; GM-CSF: investigator's brochure and supplement for intraperitoneal administration). A SUSAR will be reported to the sponsor immediately by the primary investigator, so that it can be reported electronically[60] to the Danish Medicines Authority within 7 days if the occurrence was fatal or life-threatening and within 15 days for all other occurrences. Further, a follow-up will be sent within 8 days. The sponsor will also report any SUSARs to the local Health Research Ethics Committee.

Pros and cons for the participants of the trial

The enrolled and included participants of the trial will receive the standard care given to patients with uncomplicated appendicitis at the Department of Surgery, Herlev Hospital, apart from the intravenous antibiotic treatment. If included, they will receive the necessary antimicrobial cover given during emergency surgery. However, the combination and route of administration of the drugs are new and have not been used before. A possible adverse consequence is that the new treatment is less effective than the standard clinical care. A possible positive consequence is that the new treatment is even more effective than the standard clinical care. Those, who are enrolled in the trial and subsequently excluded during the surgery, will receive the standard intravenous antibiotic treatment.

Participants of the main trial: Safety will be subject to increased surveillance during and after the surgery through direct contact with the primary investigator and repeated measurements of vital values. Thus, if any complications arise from the uncomplicated appendicitis itself, the surgery, or the trial treatment, these will be attended to immediately by a doctor, which differs from the standard care. The participant will have an extra blood sample drawn to repeat the standard panel of blood samples 4 hours after the trial treatment. This requires a total of 20.5 ml blood. The participants are required to visit the hospital 10 days after their surgery for a meeting with and an objective

examination by the primary investigator. The participant will normally see their general practitioner 10 days after the surgery to have stitches removed. However, the patients are offered to have the stitches removed during the visit by the primary investigator.

Participants in the sub-trial: Pharmacokinetics will have more blood samples drawn than usual. These blood samples will either be drawn from an intravenous access or by separate venepuncture. This will result in a few seconds of discomfort, when the needle is placed in the vein. The baseline blood sample will be drawn under full anaesthesia. Each blood sample requires 3-4 ml of blood, resulting in a total volume of 28 ml of blood. A well-known risk of blood sample collection is a hematoma.

Patients participating in both trials will in total have less than 50 ml blood drawn for extra blood samples. This is comparable to around 11% of the amount donated through a single donation of blood (450 ml) and will not have any adverse effects in otherwise healthy men.

Data monitoring and management

Access to relevant documents

The primary investigator permits direct access to source data or documents, including medical record for the purpose of monitoring, auditing, and/or inspection from the Danish Medicines Authority, GCP monitor or other regulatory authorities.

Quality control and assurance

The present procedures for quality control and assurance will be followed according to the GCP guidelines through planned and systematic agreements on monitoring before, during, and after the trial. A written authority for a third party (e.g. GCP monitor, the Danish Medicines Authority, etc.) for the access to medical records will be collected together with the informed, written consent.

Data management

Approval from the Danish Data Protection Agency will be obtained according to the Act on Processing of Personal Data (*Persondataloven*) before the inclusion of participants. Data will be prospectively registered in paper CRFs. These are prepared before the inclusion starts. CRFs are kept under lock in a closet that only the primary investigator has access to. They are kept for 10 years after the trial whereupon they will be destroyed. Subsequently the data will be stored electronically in a database. No personally identifiable data will be published. The anonymity of all participants will be secured and data kept confidential.

Ethics

Ethical considerations

The trial will be conducted according to the Helsinki II Declaration and will be approved by the local Health Research Ethics Committee before the initiation of the trial.

This trial is conducted to improve the treatment options for patients suffering from peritonitis. The trial investigates a combination of drugs that is administered intraperitoneally. This requires an access to the abdominal cavity. Healthy volunteers are thus not suitable for this trial. This pilot trial is carried out in the healthiest patients possible in whom we have intraperitoneal access: patients with uncomplicated appendicitis.

We hope that the treatment will eventually have wider implications. Currently, effective treatment options for seriously ill patients with severe peritonitis (e.g. due to perforation of the colon) are limited. The intraperitoneal combination of drugs could potentially decrease these patients' risk of complications.

Enrolment of participants

Patients who are eligible for enrolment will be personally addressed and informed about the study by the surgeon planning their surgery and by the primary investigator. The patients are fully awake and conscious. The patient

will receive both verbal and written information. Further, the patients will be informed that:

- It is an enquiry to participate in the study
- It will not influence the treatment if the patient does not wish to participate
- The information can be given later so a companion can arrive before they make a decision
- The patient has the right to time for consideration

Because of the acute nature of the disease, the patient's time for consideration last at least until the patient is taken to the operating theatre. This varies from a few hours to half a day depending on the acute surgical schedule, in which patients with acute appendicitis are given priority. Hence we cannot ensure 24 hours for consideration.

The information will be given in a room without any disturbances and where the patient can sit or lie comfortably. The patients will be given enough time to read and listen to the information and ask questions. If patients wish to have more information regarding the trial or have any questions, they are welcome to contact the primary investigator.

Finances and insurance

Financing

The sponsor and investigators from Herlev Hospital have taken the initiative for the main trial: Safety and the sub-trial: Pharmacokinetics. The funding of the main trial and sub-trial is paid through grant applications. Reponex Pharmaceuticals ApS has filed a patent application for the intraperitoneal administration of the trial drugs. They will donate an unrestricted grant for the main trial: Safety and sub-trial: Pharmacokinetics of 104,748.70 DDK to the Department of Surgery, Herlev Hospital. It will be administered at a research account by the hospital. All costs will be fully transparent.

Insurance

Patients will be covered by the Danish Act on the Right to Complain and Receive Compensation within the Danish Health Service (*Patientsforsikringsloven*), which covers patients treated in Danish hospitals. This is standard practice for Danish investigator-initiated trials.

Completion of the trial and publication

The sponsor will contact the Danish Medicines Authority no later than 90 days after the last participant has completed the main trial: Safety and the sub-trial: Pharmacokinetics.

Publication

The main trial: Safety and the sub-trial: Pharmacokinetics will be submitted separately for publication. "Positive", "negative", and inconclusive results will be submitted for publication or made public. The sub-trial: Pharmacokinetic will follow the reporting guidelines of clinical pharmacokinetic studies, ClinPK.[61]

The determination of authorship is based on the authorship criteria and recommendations of the International Committee of Medical Journal Editors (ICMJE). Authors have to fulfil all four criteria:

1. Substantial contributions to: the conception or design of the work; or the acquisition, analysis, or interpretation of data for the work;
2. Drafting the work or revising it critically for important intellectual content;
3. Final approval of the version to be published;
4. Agreement to be accountable for all aspects of the work in ensuring that the questions related to the accuracy or integrity of any part of the work are appropriately investigated and resolved.

The authors for the publication of the main trial: Safety are: Siv Fonnes, Barbara Holzknecht, Magnus Arpi, and Jacob Rosenberg. The authors for the publication of the sub-trial: Pharmacokinetics are: Siv Fonnes, Johan Juhl Weisner, Barbara Holzknecht, Magnus Arpi, and Jacob Rosenberg. Further authors may be added if they fulfil the ICMJE criteria.

Summary

Background: In secondary peritonitis, bacteria that have escaped from abdominal organs cause an infection in the abdominal cavity. Peritonitis can have long-term consequences for the patients. The severity of the disease depends on the extent of inflammation. Current treatment practice is surgical intervention (source control) in combination with the administration of intravenous antibiotics. We wish to administer two antimicrobial agents: fosfomycin and metronidazole, together with granulocyte-macrophage colony-stimulating factor (GM-CSF), which stimulates the function of macrophages. The surgical intervention is kept. The trial drugs are administered at the site of the disease: intraperitoneal administration. Thereby the abdominal cavity receives highest concentration of the drugs. Furthermore, in addition to the antimicrobial treatment, the patient's local immune defence is stimulated by GM-CSF.

This pilot trial will be conducted in otherwise healthy patients suffering from uncomplicated appendicitis. The administration of the trial treatment demands an intra-abdominal access, which is routinely established during surgery. The trial will consist a main trial: Safety and a sub-trial: Pharmacokinetics that involve and recruit from the same trial population.

Main trial: Safety: The primary objective is to investigate if intraperitoneal administration of fosfomycin, metronidazole, and GM-CSF is safe, on the basis of white blood cell counts four hours after the surgery. Secondary objectives are measurements of vital signs at baseline, 5, 10 and 15 minutes and again 4 and 12 hours after administration of the trial treatment, length of stay and side effects evaluated by an objective examination and questions about changes 12 hours and 10 days after administration of the trial treatment. A follow-up is conducted 30 days after the surgery. Fourteen otherwise healthy males ≥18 years diagnosed with uncomplicated appendicitis are needed to complete the main trial.

Sub trial: Pharmacokinetics: The primary objective is to investigate the plasma concentrations of fosfomycin after intraperitoneal administration over time. Secondary objectives are investigations of metronidazole concentrations after intraperitoneal administration over time and microbiologic investigations of

specimens from the removed appendices and/or excess abdominal fluid. Blood samples will be collected at baseline, ½, 1, 2, 4, 8, 12, and 24 hours after surgery. The collected blood samples will be stored in a biobank for 1 year. Eight otherwise healthy males ≥18 years diagnosed with uncomplicated appendicitis are needed to complete the sub-trial.

Ethics: The trial will be conducted according to the Helsinki II Declaration and after approval from both the Danish Medicines Authority and local Health Research Ethics Committee. An informed written consent will be collected from the participants prior to inclusion in the trial. Data will be monitored by the GCP-unit for hospitals of the University of Copenhagen and will be stored with approval from the Danish Data Protection Agency.

This pilot trial is conducted in patients with uncomplicated appendicitis. These patients are fully awake and conscious at inclusion and have few complications. They are often ready for discharge some hours after surgery, when the appendix is removed and their disease is cured. Because the intraperitoneal administration of drugs requires an entrance to the abdominal cavity, healthy volunteers are not suitable for this pilot trial.

Supplements

Indices	Task	Score	Scoring
Activity	Able to move four extremities voluntarily or on command	2	
	Able to move two extremities voluntarily or on command	1	
	Unable to move extremities voluntarily or on command	0	
Respiration	Able to breathe deeply and cough freely	2	
	Dyspnoea, limited breathing or tachypnoea	1	
	Apnoeic or on mechanical ventilator	0	
Circulation	BP ± 20% of pre-anaesthetic level	2	
	BP ± 20-49% of pre-anaesthetic level	1	
	BP ±50% of pre-anaesthetic level	0	
Consciousness	Fully awake	2	
	Arousable on calling	1	
	Not responding	0	
O_2 Saturation	Able to maintain O_2 saturation >92% on room air	2	
	Needs O_2 inhalation to maintain O_2 saturation >90%	1	
	O_2 saturation <90% even with O_2 supplement	0	
Dressing	Dry and clean	2	
	Wet but marked and not increasing	1	
	Growing area of wetness	0	
Pain	Pain free	2	
	Mild pain handled by oral medication	1	
	Severe pain requiring parenteral medication	0	
Ambulation	Able to stand up and walk straight*	2	
	Vertigo when erect	1	
	Dizziness when supine	0	
Fasting-Feeding	Able to drink fluids	2	
	Nauseated	1	
	Nausea and vomiting	0	
Urine Output	Has voided	2	
	Unable to void but comfortable	1	
	Unable to void and uncomfortable	0	
Total			

Table 2. After the Postanesthetic Recovery Score for Patients Having Anesthesia on Ambulatory Basis (PARSAP).[48] A score of 18 or higher defines when the patient is ready for discharge. Abbreviations: BP, blood pressure. *May be substituted by Romberg's test, or picking up 12 clips in one hand.

Type	Sample		Reference range
Blood and coagulation:	Haemoglobin		8,3-10,5 mmol/l
	Iron		9-34 µmol/l
	Transferrin		24,0-41,0 µmol/l
	Thrombocyte		$145\text{-}390{*}10^9$/l
	Clotting factor II+VII+X [INR]		2,0-3,0
	Clotting factor II+VII+X		>63 %
Infection markers:	White blood cell counts		3,5-8,8 $*10^9$/l
	White blood cell differential count:		
	Basophil		0,0-0,10 $*10^9$/l
	Eosinophil		0,04-0,50 $*10^9$/l
	Lymphocyte		1,0-3,5 $*10^9$/l
	Monocyte		0,20-0,76 $*10^9$/l
	Neutrophil		1,6-5,9 $*10^9$/l
Kidney function tests:	Urea	18-49 years:	3,2-8,1 mmol/l
		50-125 years:	3,5-8,1 mmol/l
	Creatinine		60-105 µmol/l
	Estimated glomerular filtration rate	18-29 years:	72-168 ml/min
		30-49 years:	48-150 ml/min
		49-69 years:	42-126 ml/min
Electrolytes:	Potassium		3,5-4,6 mmol/l
	Sodium		137-144 mmol/l
Liver function tests:*	Albumin	18-39 years:	36-48 g/l
		40-69 years:	36-45 g/l
	Alanine transaminase [ALAT]		10-70 U/l
	Bilirubin		5-25 µmol/l
	Alkaline phosphatase		35-105 U/l
Others:	Lactate dehydrogenase		105-205 U/l
	Amylase		25-125 U/l
	Glucose		4,2-6,3 mmol/l
	Calcium-ion free		1,18-1,32 mmol/l
	Calcium		1,18-1,32 mmol/l

Table 3. A list of the standard panel of blood samples during admission to Department of Surgery, Herlev Hospital.* "Liver function tests" also includes INR, which is listed under "Blood and coagulation".

References

1. Wittmann DH, Schein M, Condon RE. Management of secondary peritonitis. Ann Surg 1996;224:10–8.
2. Malangoni MA, Inui T. Peritonitis - the Western experience. World J Emerg Surg 2006;1:25.
3. Lieschke GJ, Burgess AW. Granulocyte colony-stimulating factor and granulocyte-macrophage colony-stimulating factor (1). N Engl J Med 1992;327:28–35.
4. Metcalf D. Control of granulocytes and macrophages: molecular, cellular, and clinical aspects. Science 1991;254:529–33.
5. Liles WC, Voorhis WC Van. Review: nomenclature and biologic significance of cytokines involved in inflammation and the host immune response. J Infect Dis 1995;172:1573–80.
6. Hercus TR, Broughton SE, Ekert PG, Ramshaw HS, Perugini M, Grimbaldeston M, Woodcock JM, Thomas D, Pitson S, Hughes T, D'Andrea RJ, Parker MW, Lopez AF. The GM-CSF receptor family: mechanism of activation and implications for disease. Growth Factors 2012;30:63–75.
7. Weisbart RH, Gasson JC, Golde DW. Colony-stimulating factors and host defense. Ann Intern Med 1989;110:297–303.
8. Burgess AW, Begley CG, Johnson GR, Lopez AF, Williamson DJ, Mermod JJ, Simpson RJ, Schmitz A DJ. Purification and properties of bacterially synthesized human granulocyte-macrophage colony stimulating factor. - PubMed - NCBI. Blood 1987;69:43–51.
9. Cantrell MA, Anderson D, Cerretti DP, Price V, McKereghan K, Tushinski RJ, Mochizuki DY, Larsen A, Grabstein K, Gillis S. Cloning, sequence, and expression of a human granulocyte/macrophage colony-stimulating factor. Proc Natl Acad Sci U S A 1985;82:6250–4.
10. Wong GG, Witek JS, Temple PA, Wilkens KM, Leary AC, Luxenberg DP, Jones SS, Brown EL, Kay RM, Orr EC. Human GM-CSF: molecular cloning of the complementary DNA and purification of the natural and recombinant proteins. Science 1985;228:810–5.
11. Selgas R, Fernández de Castro M, Jiménez C, Cárcamo C, Contreras T, Bajo MA, Vara F, Corbí A. Immunomodulation of peritoneal macrophages by granulocyte-macrophage colony-stimulating factor in humans. Kidney Int 1996;50:2070–8.
12. Toner GC, Gabrilove JL, Gordon M, Crown J, Jakubowski AA, Meisenberg B, Sheridan C, Boone T, Vincent ME MM. Phase I trial of intravenous and intraperitoneal administration of granulocyte-macrophage colony-stimulating factor. J Immunother Emphasis Tumor Immunol 1994;15:59–66.
13. Orozco H, Arch J, Medina-Franco H, Pantoja JP, González QH, Vilatoba M, Hinojosa C, Vargas-Vorackova F, Sifuentes-Osornio J. Molgramostim (GM-CSF) associated with antibiotic treatment in nontraumatic abdominal

sepsis: a randomized, double-blind, placebo-controlled clinical trial. Arch Surg 2006;141:150–3; discussion 154.

14. Presneill JJ, Harris T, Stewart AG, Cade JF, Wilson JW. A randomized phase II trial of granulocyte-macrophage colony-stimulating factor therapy in severe sepsis with respiratory dysfunction. Am J Respir Crit Care Med 2002;166:138–43.

15. Raz R. Fosfomycin: An old-new antibiotic. Clin Microbiol Infect 2012;18:4–7.

16. Kahan FM, Kahan JS, Cassidy PJ, Kropp H. The mechanism of action of fosfomycin (phosphonomycin). Ann N Y Acad Sci 1974;235:364–86.

17. Zeitlinger MA, Sauermann R, Traunmüller F, Georgopoulos A, Müller M, Joukhadar C. Impact of plasma protein binding on antimicrobial activity using time-killing curves. J Antimicrob Chemother 2004;54:876–80.

18. Joukhadar C, Klein N, Dittrich P, Zeitlinger M, Geppert A, Skhirtladze K, Frossard M, Heinz G, Müller M. Target site penetration of fosfomycin in critically ill patients. J Antimicrob Chemother 2003;51:1247–52.

19. Kuemmerle HP, Murakawa T, Santis F De. Pharmacokinetic evaluation of fosmidomycin, a new phosphonic acid antibiotic. Chemioterapia 1987;6:113–9.

20. Goto M, Sugiyama M, Nakajima S, Yamashina H. Fosfomycin kinetics after intravenous and oral administration to human volunteers. Antimicrob Agents Chemother 1981;20:393–7.

21. Potel G, Meignier M, Baron D, Reynaud A, Touze MD, Courtieu AL. Pharmacokinetics of fosfomycin in normal and burn patients. Effect of probenecid. Drugs Exp Clin Res 1989;15:177–84.

22. Cadórniga R, Diaz Fierros M, Olay T. Pharmacokinetic study of fosfomycin and its bioavailability. Chemotherapy 1977;23 Suppl 1:159–74.

23. Falagas ME, Kastoris AC, Kapaskelis AM, Karageorgopoulos DE. Fosfomycin for the treatment of multidrug-resistant, including extended-spectrum beta-lactamase producing, Enterobacteriaceae infections: a systematic review. Lancet Infect Dis 2010;10:43–50.

24. Zykov IN, Sundsfjord A, Småbrekke L, Samuelsen Ø. The antimicrobial activity of mecillinam, nitrofurantoin, temocillin and fosfomycin and comparative analysis of resistance patterns in a nationwide collection of ESBL-producing Escherichia coli in Norway 2010-2011. Infect Dis (London, England) 2015:1–9.

25. Lu C-L, Liu C-Y, Huang Y-T, Liao C-H, Teng L-J, Turnidge JD, Hsueh P-R. Antimicrobial susceptibilities of commonly encountered bacterial isolates to fosfomycin determined by agar dilution and disk diffusion methods. Antimicrob Agents Chemother 2011;55:4295–301.

26. Falagas ME, Roussos N, Gkegkes ID, Rafailidis PI, Karageorgopoulos DE. Fosfomycin for the treatment of infections caused by Gram-positive cocci with advanced antimicrobial drug resistance: a review of microbiological, animal and clinical studies. Expert Opin Investig Drugs 2009;18:921–44.

27. Falagas ME, Giannopoulou KP, Kokolakis GN, Rafailidis PI. Fosfomycin: use beyond urinary tract and gastrointestinal infections. Clin Infect Dis 2008;46:1069–77.

28. Tobudic S, Matzneller P, Stoiser B, Wenisch JM, Zeitlinger M, Vychytil A, Jaeger W, Boehmdorfer M, Reznicek G, Burgmann H. Pharmacokinetics of intraperitoneal and intravenous fosfomycin in automated peritoneal dialysis patients without peritonitis. Antimicrob Agents Chemother 2012;56:3992–5.

29. Bouchet JL, Albin H, Quentin C, Barbeyrac B de, Vincon G, Martin-Dupont P, Potaux L, Aparicio M. Pharmacokinetics of intravenous and intraperitoneal fosfomycin in continuous ambulatory peritoneal dialysis. Clin Nephrol 1988;29:35–40.

30. http://www.produktresume.dk/docushare/dscgi/ds.py/View/Collection-96.

31. Lamp KC, Freeman CD, Klutman NE, Lacy MK. Pharmacokinetics and pharmacodynamics of the nitroimidazole antimicrobials. Clin Pharmacokinet 1999;36:353–73.

32. Schwartz DE, Jeunet F. Comparative pharmacokinetic studies of ornidazole and metronidazole in man. Chemotherapy 1976;22:19–29.

33. Taylor JA, Migliardi JR, Wittenau MS Von. Tinidazole and metronidazole pharmocokinetics in man and mouse. Antimicrob Agents Chemother 1969;9:267–70.

34. Saha SK. Peritoneal lavage with metronidazole. Surg Gynecol Obstet 1985;160:335–8.

35. Saha SK. Efficacy of metronidazole lavage in treatment of intraperitoneal sepsis. A prospective study. Dig Dis Sci 1996;41:1313–8.

36. el-Sefi TA, El-Awady HM, Shehata MI, Al-Hindi MA. Systemic plus local metronidazole and cephazolin in complicated appendicitis: a prospective controlled trial., 1989.

37. Andåker L, Höjer H, Kihlström E, Lindhagen J. Stratified duration of prophylactic antimicrobial treatment in emergency abdominal surgery. Metronidazole-fosfomycin vs. metronidazole-gentamicin in 381 patients., 1987.

38. Andåker L, Burman LG, Eklund A, Graffner H, Hansson J, Hellberg R, Höjer H, Ljungqvist U, Kjellgren K, Kling PA. Fosfomycin/metronidazole compared with doxycycline/metronidazole for the prophylaxis of infection after elective colorectal surgery. A randomised double-blind multicentre trial in 517 patients., 1992.

39. Lieschke GJ, Maher D, O'Connor M, Green M, Sheridan W, Rallings M, Bonnem E, Burgess AW, McGrath K, Fox RM. Phase I study of intravenously administered bacterially synthesized granulocyte-macrophage colony-stimulating factor and comparison with subcutaneous administration. Cancer Res 1990;50:606–14.

40. Hovgaard D, Mortensen BT, Schifter S, Nissen NI. Comparative

pharmacokinetics of single-dose administration of mammalian and bacterially-derived recombinant human granulocyte-macrophage colony-stimulating factor. Eur J Haematol 1993;50:32–6.

41. Lieschke GJ, Maher D, Cebon J, O'Connor M, Green M, Sheridan W, Boyd A, Rallings M, Bonnem E, Metcalf D. Effects of bacterially synthesized recombinant human granulocyte-macrophage colony-stimulating factor in patients with advanced malignancy. Ann Intern Med 1989;110:357–64.

42. http://www.eucast.org/fileadmin/src/media/PDFs/EUCAST_files/Rationale_documents/Fosfomycin_rationale_1.0_20130203.pdf.

43. Yamamoto M, Pop-Vicas AE. Treatment for infections with carbapenem-resistant Enterobacteriaceae: what options do we still have? Crit Care 2014;18:229.

44. Piraino B, Bailie GR, Bernardini J, Boeschoten E, Gupta A, Holmes C, Kuijper EJ, Li PK-T, Lye W-C, Mujais S, Paterson DL, Fontan MP, Ramos A, Schaefer F, Uttley L. Peritoneal dialysis-related infections recommendations: 2005 update. Perit Dial Int 25:107–31.

45. Li PK-T, Szeto CC, Piraino B, Bernardini J, Figueiredo AE, Gupta A, Johnson DW, Kuijper EJ, Lye W-C, Salzer W, Schaefer F, Struijk DG. Peritoneal dialysis-related infections recommendations: 2010 update. Perit Dial Int 30:393–423.

46. Nikitidou O, Liakopoulos V, Kiparissi T, Divani M, Leivaditis K, Dombros N. Peritoneal dialysis-related infections recommendations: 2010 update. What is new? Int Urol Nephrol 2012;44:593–600.

47. Ballinger AE, Palmer SC, Wiggins KJ, Craig JC, Johnson DW, Cross NB, Strippoli GFM. Treatment for peritoneal dialysis-associated peritonitis. Cochrane database Syst Rev 2014;4:CD005284.

48. Aldrete JA. Modifications to the postanesthesia score for use in ambulatory surgery. J Perianesth Nurs 1998;13:148–55.

49. http://www.medicines.org.uk/emc/glossary?view=130.

50. Mayama T, Yokota M, Shimatani I, Ohyagi H. Analysis of oral fosfomycin calcium (Fosmicin) side-effects after marketing. Int J Clin Pharmacol Ther Toxicol 1993;31:77–82.

51. Michalopoulos A, Virtzili S, Rafailidis P, Chalevelakis G, Damala M, Falagas ME. Intravenous fosfomycin for the treatment of nosocomial infections caused by carbapenem-resistant Klebsiella pneumoniae in critically ill patients: A prospective evaluation. Clin Microbiol Infect 2010;16:184–6.

52. Corti N, Sennhauser FH, Stauffer UG, Nadal D. Fosfomycin for the initial treatment of acute haematogenous osteomyelitis. Arch Dis Child 2003;88:512–6.

53. Michalopoulos AS, Livaditis IG, Gougoutas V. The revival of fosfomycin. Int J Infect Dis 2011;15:e732–9.

54. Roussos N, Karageorgopoulos DE, Samonis G, Falagas ME. Clinical significance of the pharmacokinetic and pharmacodynamic characteristics

of fosfomycin for the treatment of patients with systemic infections. Int J Antimicrob Agents 2009;34:506–15.

55. Nøhr M, Andersen JC, Juul-Jensen KE. Prophylactic single-dose fosfomycin and metronidazole compared with neomycin, bacitracin, metronidazole and ampicillin in elective colorectal operations., 1990.

56. Cebon J, Lieschke GJ, Bury RW, Morstyn G. The dissociation of GM-CSF efficacy from toxicity according to route of administration: a pharmacodynamic study. Br J Haematol 1992;80:144–50.

57. Lieschke GJ, Cebon J, Morstyn G. Characterization of the clinical effects after the first dose of bacterially synthesized recombinant human granulocyte-macrophage colony-stimulating factor. Blood 1989;74:2634–43.

58. Meisel C, Schefold JC, Pschowski R, Baumann T, Hetzger K, Gregor J, Weber-Carstens S, Hasper D, Keh D, Zuckermann H, Reinke P, Volk H-D. Granulocyte-macrophage colony-stimulating factor to reverse sepsis-associated immunosuppression: a double-blind, randomized, placebo-controlled multicenter trial. Am J Respir Crit Care Med 2009;180:640–8.

59. Barthelsson C, Sandblom G, Ljesevic-Nikoletic S, Hammarqvist F. Effects of Intra-abdominally Instilled Isotonic Saline on Pain, Recovery, and Health-Related Quality-of-Life Following Laparoscopic Cholecystectomy: A Randomized Prospective Double-Blind Controlled Study. World J Surg 2015;39:1413–20.

60. http://sundhedsstyrelsen.dk/da/medicin/regulering/kliniske-forsoeg/bivirkninger/indberetning-af-mistaenkte-uventede-og-alvorlige-bivirkninger-susar-set-i-kliniske-forsoeg-e-blanket.aspx.

61. Kanji S, Hayes M, Ling A, Shamseer L, Chant C, Edwards DJ, Edwards S, Ensom MHH, Foster DR, Hardy B, Kiser TH, Porte C la, Roberts JA, Shulman R, Walker S, Zelenitsky S, Moher D. Reporting Guidelines for Clinical Pharmacokinetic Studies: The ClinPK Statement. Clin Pharmacokinet 2015.
